# *Glycosmis pentaphylla* (Rutaceae): A Natural Candidate for the Isolation of Potential Bioactive Arborine and Skimmianine Compounds for Controlling Multidrug-Resistant *Staphylococcus aureus*

**DOI:** 10.3389/fpubh.2020.00176

**Published:** 2020-06-10

**Authors:** Natarajan Murugan, Ramalingam Srinivasan, Athiappan Murugan, Myunghee Kim, Devarajan Natarajan

**Affiliations:** ^1^Natural Drug Research Laboratory, Department of Biotechnology, School of Biosciences, Periyar University, Salem, India; ^2^Department of Food Science and Technology, Yeungnam University, Gyeongsan-si, South Korea; ^3^Department of Biotechnology, K. S. Rangasamy College of Arts and Science, Namakkal, India; ^4^Department of Microbiology, School of Biosciences, Periyar University, Salem, India

**Keywords:** *Gycosmis pentaphylla*, antibacterial compounds, chromatography, spectral study, MDR *Staphylococcus aureus*, arborine, skinmmianine

## Abstract

Several multidrug-resistant organisms have emerged, which increases the threat to public health around the world and a limited number of therapeutics were available to counteract these issues. Thus, researchers are trying to find out novel antimicrobials to overcome multidrug-resistant issues. The present study aimed to isolate antibacterial principles against the clinical isolates of multidrug-resistant (MDR) *Staphylococcus aureus* from the ethyl acetate extract of *Gycosmis pentaphylla*. The isolation and structural characterization of bioactive compounds were carried out using various chromatographic techniques (TLC, column, HPLC, and LC-MS) and spectral studies such as FT-IR, CHNS analysis, ^1^H-NMR, and ^13^C-NMR. The antimicrobial potential of isolated compounds was assessed according to the standard methods. The isolated compounds were identified as arborine and skimmianine, which exhibited a significant antibacterial effect with the lowest MIC and MBC values against MDR *S. aureus* and *in vitro* kinetic and protein leakage assays supported the antimicrobial activity. Significant morphological changes such as uneven cell surfaces and morphology, cell shrinkage, and cell membrane damages were observed in the MDR *S. aureus* upon the treatment of arborine and skimmianine. The present investigation concludes that the isolated arborine and skimmianine compounds from *G. pentaphylla* harbor a strong antibacterial activity against MDR *S. aureus* and may be used as alternative natural drugs in the treatment of MDR *S. aureus*.

## Introduction

*Staphylococcus aureus* is a Gram-positive bacterium that belongs to the family Staphylococaceae and is often found at a commensal on the skin, skin glands, and mucous membranes, particularly in the nose of a healthy individual (1). It is causing infections ranging from relatively mild skin and soft tissue infections to life-threatening sepsis, pneumonia, osteomyelitis, endocarditis, as well as toxin-mediated syndromes such as toxic shock syndrome and food poisoning (2). Multidrug resistant *S. aureus* (MRSA) is resistant to two or more antimicrobial agents like penicillin, oxacillin, ampicillin, and methicillin. In general, MRSA infections have been categorized into four groups based on their sources: healthcare-associated hospital-onset MRSA (HAHO-MRSA), community-associated MRSA (CA-MRSA), healthcare-associated MRSA with community-onset (HACO-MRSA), and livestock-associated MRSA (LA-MRSA) (3). Although MRSA infections mainly occur in hospitals, the human illness caused by community-associated MRSA (CA-MRSA) is increasing considerably (4).

In recent years, drug-resistant human pathogenic bacteria have been frequently reported (5). The drug-resistant Enterotoxigenic *Escherichia coli* pathogenic bacteria demand the development of new antimicrobial drugs with a novel mode of action, targeting either the cell membrane or intracellular targets (6). Increasing the bacterial resistance is prompting a resurgence in the research on the antimicrobial role of herbs available in nature as effective combinations against antibiotics-resistant bacteria (7). Moreover, using natural products also help to diminish the toxicity of the drugs when they are used on humans (8).

Since ancient times, plants have been the primary sources of many therapeutic agents that possess several secondary metabolites with significant physiological effects. Recently, many active ingredients have been isolated from plants and those are used to develop synthetic analogs for the treatment of ailments. There are 300 plant species frequently used in 7,800 drug-manufacturing units around India, which consume more than 2,000 tons of herbs annually (9). Generally, bioactive principles are isolated from different parts of plants such as leaves, bark, roots, seeds, etc. based on folklore usage. According to WHO, medicinal plants would be the best source to obtain a variety of drugs (10), and ~20% of the known plants were subjected to biological tests and a sustainable number of new antibiotics were introduced into the market (11). Concurrently, the characterizations of antimicrobial compounds from medicinal plants have been very challenging to the researchers (12). Hence, a systemic screening of plants for the identification of antimicrobial compounds to act against microbial pathogens needs an hour. Recently, many researchers have been focused on the isolation of potential bioactive compounds from medicinal plants to produce high-quality and potential secondary metabolites responsible for the control of microorganisms (13, 14). With this background, the present study was aimed to isolate antibacterial principles from *Glycosmis pentaphylla*.

## Materials and Methods

### Isolation and Identification of MDR *S. aureus*

About 500 specimens [wound (138), pus (122), blood (119), sputum (70), and urine (51)] were collected from patients suspected to have *S. aureus* infections from private and government hospitals in and around Salem and Namakkal Districts, Tamil Nadu, India. The present study has ethical clearance from the institutional ethical committee (reference no.: PU/IEC/HR/2014/008 dated: 31/06/2014), Periyar University, Salem, Tamil Nadu. The methods of isolation and identification of the selected MDR *S. aureus* strains were described in our previous published data (15, 16). The isolation and identification of MDR *S. aureus* were carried out by the standard methods such as colony morphology on differential media, microscopic observations, and biochemical tests, and confirmed by molecular analysis like 16s rRNA sequencing. The antibiotic-resistant potential of isolated *S. aureus* was identified using the standard antimicrobial susceptibility test and identification of antibiotic-resistant marker genes such as *MecA, blaZ, Aph (III)*, etc. The 16s rRNA sequence of isolated MDR *S. aureus* (101, 270, 315, 319, and 410) strains used in the present study is deposited in GenBank and GenBank accession nos. are KU198419 (*S. aureus* 101), KX290715.1 (*S. aureus* 270), KX454514 (*S. aureus* 315), KX447584 (*S. aureus* 319), and KX447585 (*S. aureus* 410).

### Plant Material and Extraction

**Figure 1:**
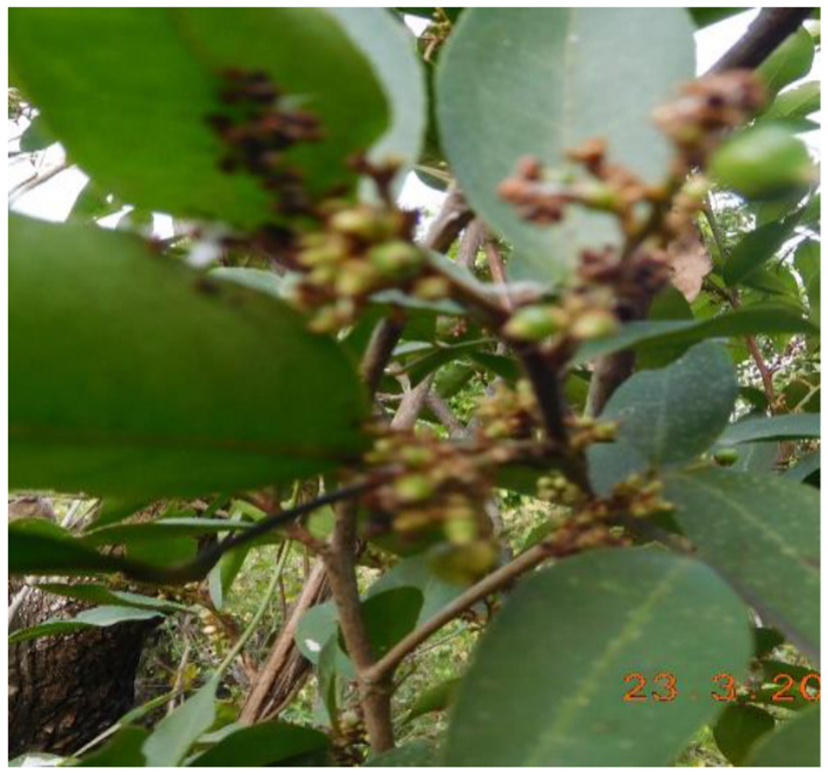
A close view of *Glycosmis pentaphylla* (Rutaceae).

The healthy and young leaves of *G. pentaphylla* (Figure 1) were collected from Vellimalai village (Latitude 11° 47′ 55.6836″ N, longitude 78° 41′ 58.0056″ E), Villupuram district, Tamil Nadu, India. The taxonomic identification of collected plant material was done by the Botanical Survey of India (Southern Circle), Tamil Nadu Agricultural University, Coimbatore, Tamil Nadu, India (reference letter No BSI/SRC/5/26/2016/402). The voucher specimen of collected plant material was deposited (No. PU/BT/NDRL/2016/09) in the Natural Drug Research Laboratory, Department of Biotechnology, Periyar University, Salem, Tamil Nadu, India. The collected plant materials were washed twice in running tap water and shade dried at room temperature for 3 weeks. The air-dried plant leaves were pulverized, using an electric blender to make a fine powder. A total of 3 kg of powdered *G. pentaphylla* leaves was sequentially extracted with different organic solvents (1:10 solvent ratio) in an increasing polarity (hexane, chloroform, ethyl acetate, acetone, and methanol) using a Soxhlet apparatus until the efflux solvents become colorless. All extracts were filtered through filter paper (Whatman No. 1) and dried under vacuum at 40°C. The dried crude solvent extracts were stored in a freezer at −4°C for further study (17).

### Isolation of Antibacterial Principles

The preliminary antibacterial activity results of various crude extracts of *G. pentaphylla* (15) revealed that the crude ethyl acetate of *G. pentaphylla* possesses a significant high antibacterial potential; thus, the ethyl acetate extract was selected for isolation of active principles. The selected ethyl acetate extract was subjected to different chromatography techniques and the structure of isolated bioactive compounds were identified using various spectral studies.

### Separation of Active Compounds by Chromatography

The ethyl acetate extracts (in both low and high concentration) were spotted on the TLC plate (TLC Silica gel F254, Merck, Germany, 10 × 10 cm^2^) origin and kept in a TLC chamber containing the desired solvent system. The solvent traveled to the top of the TLC plate by capillary action until it reached the solvent front. The TLC plate was taken out of the solvent tank. The dried TLC plate was viewed by iodine vapor and visualized under UV light (low and high wavelength). The same method was followed to find the optimal solvent system with other TLC plates in various solvent systems like hexane:ethyl acetate, ethyl acetate:methanol, chloroform:ethyl acetate, and chloroform:methanol in 100:0 to 0:100 ratios by increasing 10% more polar solvents used in the solvent system until a good resolution was noticed (18). The selected bioactive extract of *G. pentaphylla* was subjected to column chromatography to obtain fractions by increasing polarity of eluents using different solvent systems (chosen from the TLC trials) of chloroform:methanol 100:0, 80:20, 70:30, 60:40, and 50:50 ratios by increasing 10–20% more polar solvent used in the solvent system. The column (120 × 4 cm) was packed with a solution of silica gel (60–120 mesh) with ethyl acetate using the wet slurry method. This involves preparing a solution of silica gel, with chloroform filled in the column up to one-third (about 40 cm) length. A significant amount of chloroform was added to the column and allowed to drain for silica gel setting in the column and the volume of the solvent collected was again poured back into the column.

The ethyl acetate extract (60 g) was absorbed into silica gel (100–200 mesh) by triturating in a mortar and left for 1 h to dry. The dried plant extract coated with silica gel was loaded on the prepared column by adding gently into the column filled with chloroform without any air bubbles and eluted with the desire solvent system. A total of 60 fractions was obtained and tested for their antibacterial potential against MDR *S. aureus* strains (clinical isolates) according to the method of Srinivasan et al. (19). The *G. pentaphylla* leaf ethyl acetate extract 9.7:0.3 chloroform:methanol ratio fraction (GPLCM-58F) showed significant antibacterial activity against *S. aureus* strains, which selected for further purification of bioactive compounds. The selected fraction was again monitored by TLC (pre-coated plate, 0.02 mm thick) to determine the optimal solvent system for further purification in column chromatography. Another small size column chromatography (60 × 2 cm) was employed for further purification of selected fractions as per the abovementioned procedures. The fractions were eluted with chloroform:methanol (9:1–9.8:0.2 ratio) solvent system by increasing 0.1% of the volume of the polar solvents used in the solvent system. All the collected column fractions were again examined for antimicrobial activity and purity by TLC (pre-coated plate, 0.02 mm thick) for the single spot and the *R*_f_ values were calculated.

A total of two fractions (GPSC-55SF and GPSC-73SF) exhibited considerable antibacterial activity; these fractions again were subjected to preparative TLC purification (20). The preparative TLC plate was performed based on the noted *R*_f_ values. The fractions were scraped with silica powder using the sterile scoop and the collected UV-active pure compounds were dissolved in ethyl acetate solvent, and it can allow stirring with a magnetic stirrer for 1 h. After the pure fraction of active compounds was separated in the solvent, it was collected and concentrated under vacuum conditions. The UV-active pure fractions of the compound (GP-1 and GP-2) with similar *R*_f_ value was pooled together and subjected to the antimicrobial activity and spectral studies.

### Structural Identification of Isolated Compounds

#### Liquid Chromatography–Mass Spectroscopy (LC-MS) Analysis

LC-MS analyses of the two isolated compounds were carried out according to the method of Natarajan et al. (21) using a Thermo/Finnigan Surveyor System consisting of a degasser, binary pump, autosampler, and column heater. The LC was coupled with the Ion Trap mass spectrometer (Thermofleet, LCQ-Fleet) supported with an ESI ion source. Data acquisition and mass spectrometric evaluation were carried out in a personal computer with Data Analysis software (Qual Browser; Thermo Electron, San Jose, CA). For the chromatographic separation, Acquity BEC 1.7-μm C18 column (2.1 × 50 mm) was used. The column was programmed to run 95% of 0.1% acetic acid in water and 5% acetonitrile for both compounds 1 and 2. The final, elution program was operated at the linear gradient level of acetonitrile from 100 to 5% for a minimum 2 min. The flow rate was 1 ml/min and the injection volume was 1 μl. The capillary voltage, column temperature, nebulizer pressure, and gas flow rate were set to 20 V, 300°C, 40 psi, and 15 ml/min, respectively, for the entire MS analysis.

#### Nuclear Magnetic Resonance (NMR) Spectroscopy Analysis

The structures of the isolated compounds were elucidated primarily by homonuclear (^1^D) NMR (22). ^1^D NMR experiments included ^1^H and ^13^C NMR, which used to locate atom positions and fragment units. NMR spectra were recorded on a Bruker Avance 300-MHz and/or Bruker Avance 600-MHz spectrometers coupled with Topsin acquisition software. Samples were dissolved in 500–600 μl of suitable deuterated solvent. An NMR experiment on samples with very little mass was carried out using Shigemi NMR tubes (100–200 μl sample). Signals were recorded in chemical shifts (δ) and expressed in parts per million (ppm), with coupling constants (*J*) calculated in hertz (Hz).

#### Fourier Transform Infrared Spectroscopy (FT-IR) Analysis

The UV-active pure compounds (GP-1 and GP-2) of fraction samples were used for FT-IR analysis. The dried compounds (each 3 mg) were encapsulated in 100 mg of KBr pellet, to prepare translucent sample discs. The compounds were loaded in an FTIR spectroscope (Shimadzu, Japan), measured from 400 to 4,000 cm^−1^ and at a resolution of 4 cm^−1^.

#### High-Performance Liquid Chromatography (HPLC) Analysis

The HPLC analysis of active compounds isolated from *G. pentaphylla* ethyl acetate extract was performed using the modified method of Hawry et al. (23). About 1 mg of concentrated sample was dissolved in 1 ml of chloroform:methanol (9.8:0.2) and 20 μl was injected to determine the purity of compounds.

### Antibacterial Activity of Isolated Compounds

The selected MDR *S. aureus* strain suspension culture was prepared by growing a single colony overnight in Luria-Bertani (LB) broth with a turbidity of 0.5 McFarland standards. The antibacterial activity of isolated compounds was evaluated against MDR *S. aureus* strains using the agar well diffusion method (24). The isolated compounds (10 μg/ml) were added into the corresponding wells using a micropipette. Commercial antibiotic (amoxicillin) was used as a positive control, whereas DMSO served as a negative control. The plates were incubated at 37°C for 24 h and the diameter of the growth inhibition zone was measured.

#### MIC and MBC Test

Minimum inhibitory concentration (MIC) of the isolated compounds was carried out using the microdilution method (25), using LB broth (Hi-media, India), and inoculum was adjusted to 2.5 × 10^5^ CFU/ml. In brief, 10 μl (2.5 × 10^5^ CFU/ml) of each MDR *S. aureus* strains was added individually in 1 ml of LB broth. Different concentrations (0.5, 1, and 2 μg/ml) of the isolated compounds were mixed with test tubes containing the MDR *S. aureus* strains. After 24-h incubation, the MIC values were obtained by visual observation of bacterial growth. The minimum bactericidal concentration (MBC) value of the isolated compounds was evaluated using the method of Natarajan et al. (25). The MBC values were determined by sub-culturing (10 μl) the MIC dilutions into the sterile Müller Hinton agar plates and incubated at 37°C for 24 h, and the results were observed.

### Mechanism of Action of Isolated Compounds Against MDR *S. aureus*

#### Protein Leakage Assay

The impact of the isolated compounds on the MDR *S. aureus* cells was measured in the terms of leakage of intracellular protein materials. The MDR *S. aureus* were treated with the isolated compounds at 37°C for 120 min; each cell suspension was centrifuged at 7,000 RPM. About 100 μl of each sample supernatant was mixed with 900 μl of Bradford reagent and then incubated for 10 min. The optical density was measured at 595 nm. Bovine serum albumin was used as a standard protein and the experiment was done in triplicate (26).

#### *In vitro* Killing Kinetic Assay

The time-kill kinetic assays were performed in five test tubes (two sets) containing an initial inoculum of 1 × 10^6^ CFU/ml in tryptic soy broth with isolated compounds according to the modified method of García et al. (27). Changes in the bacterial count during exposure of the isolated compounds to the bacteria were monitored in five test tubes. MDR *S. aureus* culture alone served as a control. The bacterial counts of the treated samples were determined in 3-h intervals up to 12-h periods (0, 3, 6, 9, and 12 h) of incubation. One hundred microliters of treated samples was diluted and 10 μl of each sample was spread on Baird–Parker agar plates and incubated at 37°C overnight, and the control samples were incubated under the same conditions. The number of viable cells in each tube was estimated after counting bacterial colonies on plates and by multiplying the appropriate dilution factor (28). All the experiments were conducted in triplicate, and mean values were measured.

#### SEM Analysis

The effect of isolated compounds on the morphology of MDR *S. aureus* strains was observed under scanning electron microscopy (SEM) after the bacterial cells were treated with the isolated compounds (arborine and skimmianine) for 6 h. After the treatment, 1 ml of each test bacterial strain was collected, centrifuged, and washed three times with phosphate buffer saline and incubated for 30 min at 4°C and fixed with 2.5% glutaraldehyde. The MDR *S. aureus* cells were dehydrated in ethanol, freeze-dried under vacuum condition, coated with an ion sputtering apparatus, and observed through SEM (Hitachi S-3400N). All strains of MDR *S. aureus* cells that are not exposed to the isolated compounds and a standard reference strain of *S. aureus* (MTCC 96) [procured from Microbial Type Culture Collection and Gene Bank (MTCC), Institute of Microbial Technology, Chandigarh, India] cells were similarly processed and used as controls (29).

## Results

### Isolation of Bioactive Compounds

Chloroform and methanol were used in various proportions as mobile phases. After elution, the purity of each fraction was tested by analytical TLC using chloroform:methanol in the ratio 9.7:0.3, which showed clear separation of fractions. The TLC containing nine major active compounds with different *R*_f_ values and similar *R*_f_ value fractions were scraped and pooled together (Figure S1) for further analysis. The ethyl acetate solvent extract of *G. pentaphylla* leaves (60 g) was applied to a silica gel column for isolation of the bioactive compounds. The column was eluted with a linear gradient solvent system consisting of chloroform:methanol, by increasing 10% polarity (100:0 to 0:100) of the solvents and the fractions were collected in a glass container. The similar *R*_f_ value fractions were pooled together according to the TLC profile and serially numbered (GP1, GP2, GP3, etc.) and all those fractions were kept at room temperature to allow condensation. After solvent evaporation, all collected fractions were tested for their antibacterial activity against the MDR *S. aureus* strains.

#### Spectroscopic Analysis

The structural characterization of the isolated compounds was identified using NMR, LC-MS mass spectrometry, and HPLC analysis. UV and IR spectroscopy along with the determination melting point was carried out as they required for both the physical and structural characterization of isolated compounds.

#### HPLC Analysis

The purity of isolated compounds was checked by HPLC analysis. The isolated active compound shows a separation peak at a retention time of 3.212 and 3.434 min for active compounds 1 and 2 respectively. In both the solvent system used, the purity of the active compounds was indicated as a single sharp peak (Figure S2).

### Structural Elucidation of Bioactive Compounds

#### Physical Properties of Isolated Compounds

Compound 1 (arborine) was a yellowish-green color and soluble in ethyl acetate, methyl chloride, and DMSO, and insoluble in water. The melting point of the compound was 160–161°C and the yield was 120 mg. The *R*_f_ value of compound 1 was 0.98 mm in analytical TLC using a chloroform:methanol 9.8:0.2 solvent system as the mobile phase, whereas compound 2 (skimmianine) appeared as yellow color powder, soluble in ethyl acetate, methyl chloride, and DMSO. The yield of the isolated compound was 150 mg and the melting point was 180°C. The *R*_f_ value of compound 2 was 0.5 mm in analytical TLC using a chloroform:methanol 9.8:0.2 solvent system as the mobile phase.

#### FT-IR Spectrum Analysis of Compounds

The FT-IR spectrum of arborine shows a broad peak at 1,602 cm^−1^, which corresponds to the carbonyl group. The aromatic C–H stretching peak was observed at 2,925 cm^−1^ while aliphatic C–H stretching frequency was found at 2,853 cm^−1^. The absorption band at 1,264 and 1,402 cm^−1^ corresponds to the C=N stretch of amide and CH_3_ bend, respectively. The aromatic C–H out of the plane bending vibration was detected at 765 cm^−1^ (Figure S3, Table 1). The FT-IR analysis of skimmianine showed an absorption band at 1,616 cm^−1^ due to the C=N group and characteristic phenyl ether C–O stretching vibration observed at 1,266 and 1,056 cm^−1^. The C–H stretching vibration corresponding to aromatic was observed at 3,116 cm^−1^ while aliphatic C–H stretching vibration was identified at 2,937 and 2,839 cm^−1^. The characteristic methyl bend and aromatic C–H bend out of plane vibrations were seen at 1,390 cm^−1^ and 739 cm^−1^, respectively. The stretching frequency of C–O in the furan ring was observed at 1,056 and 1,192 cm^−1^ (Figure S7, Table 1).

**Table 1:** FT-IR spectrum of arborine and skimmianine compounds.

**Compound name**	**ν_max_ (cm^−1^)**	**Functional groups**
Arborine	2,925	Aromatic C–H Stretch
	2,855	Aliphatic C–H Stretch
	1,602	C=O
	1,264	C–N Stretch
	1,402	Methyl bend
	765	Aromatic C–H bend out of the plane
Skimmianine	3,116	Aromatic C–H Stretch
	2,937, 2,839	Aliphatic C–H Stretch
	1,616	C=N Stretch
	1,266, 1,056	Phenyl ether C–O
	1,390	Methyl bend
	1,056, 1,192	Furan C–O
	736	C–H bend out of the plane

#### LC-MS and Elemental Analysis of Compounds

The mass spectra of arborine showed a molecular ion peak at m/z 251 (M+1)^+^ corresponding to the molecular formula C_16_H_14_N_2_O. The molecular weight was exactly matching with expected structure arborine (Figure S6). The result of the CHNS/O analysis of arborine showed carbon 76.22%, hydrogen 5.48%, nitrogen 10.96%, and oxygen 6.39%, which is in agreement with theoretical values (Table 2). The mass spectra of skimmianine showed a molecular ion peak at m/z 260 (M+1)^+^ corresponding to the molecular formula C_14_H_13_NO_4_. The molecular weight of the compound exactly matched the expected structure of skimmianine (Figure S10). The result of the CHNS/O analysis of skimmianine showed carbon 63.36%, hydrogen 4.98%, nitrogen 5.02%, and oxygen 24.96%, which is in agreement with theoretical values. The elemental analysis result was interpreted with molecular mass, which revealed the molecular formula of the GP-2 compound as C_14_H_13_NO_4_ (Table 2).

**Table 2:** Elemental analysis of arborine and skimmianine compounds.

**Compound name**	**Elements**	**Theoretical value (%)**	**Observed value (%)**
Arborine	Carbon	76.78	76.22
	Hydrogen	5.67	5.48
	Nitrogen	11.19	10.96
	Oxygen	6.39	6.23
Skimmianine	Carbon	64.86	63.36
	Hydrogen	5.05	4.98
	Nitrogen	5.40	5.02
	Oxygen	24.68	24.96

#### ^1^H-NMR and ^13^C-NMR Analysis of Compounds

The ^1^H-NMR spectrum of arborine revealed a total of 14 protons present in the compound. The characteristic N-methyl proton was observed at δ 3.63 ppm as a singlet. The benzylic protons present in the arborine was seen at δ 4.29 ppm as singlet (Figure S4). In the downfield aromatic region, eight aromatic protons were observed between δ 7.28 and δ 7.36 ppm as a multiplet. The quinazoline fused aromatic ring protons numbered 3, 1, and 4 were observed at δ 7.46 ppm (triplet), δ 7.71 ppm (doublet), and δ 8.37 ppm (doublet), respectively (Figure S4, Table 3).

**Table 3:** NMR (^1^H-NMR and ^13^C-NMR) spectral data of arborine compound.

**Chemical shift (δ ppm)**	**Proton/carbon numbered**	**Splitting pattern**	**Nature of the proton/carbon**
^**1**^**H-NMR**			
3.63	5	Singlet	N-methyl proton
4.29	6	Singlet	Benzylic proton
7.28–7.36	7, 8, 9, 10, 11, 12, 13, 14	Multiplet	Aromatic (benzene ring)
7.46	2, 3	Triplet	Aromatic (quinazoline ring)
7.71	1	Doublet	Aromatic (quinazoline ring)
8.37	4	Doublet	Aromatic (quinazoline ring)
^**13**^**C-NMR**			
34.8	7	Singlet	N-methyl carbon
43.4	10	Singlet	Benzylic carbon
114.4	1	Singlet	Aromatic (Quinazoline ring)
120.0	8	Singlet	Aromatic (Quinazoline ring)
125.9	3	Singlet	Aromatic (Quinazoline ring)
127.3	14	Singlet	Aromatic (Benzene ring)
128.2	13, 15	Singlet	Aromatic (Benzene ring)
128.5	4	Singlet	Aromatic (Quinazoline ring)
129.0	12, 16	Singlet	Aromatic (Benzene ring)
133.7	2	Singlet	Aromatic (Quinazoline ring)
134.5	11	Singlet	Aromatic (Benzene ring)
141.4	9	Singlet	Aromatic (Quinazoline ring)
162.2	6	Singlet	C=N (Quinazoline ring)
169.0	5	Singlet	Amide C=O

In the ^13^C-NMR of arborine, N-methyl and benzylic carbons are observed at δ 34.8 and δ 43.4 ppm, respectively. The highest carbon chemical shift δ 169.0 ppm was observed for amide carbonyl carbon. The chemical shift for imine carbon (C=N) was seen at δ 162.2 ppm. The aromatic carbon chemical shifts were observed between δ 114.4 and δ 141.4 ppm (Figure S5, Table 3). Three methoxy groups present in skimmianine showed chemical shift values between δ 4.03 and δ 4.49 ppm. All the methoxy protons in the upfield showed a clear singlet pattern. The two protons correspond to the furan ring observed at δ 7.02 and δ 7.56 ppm, respectively, and those protons show a doublet splitting pattern. The other aromatic protons are seen at δ 7.23 and δ 7.99 ppm, which also showed a doublet splitting pattern (Figure S8, Table 4). In the ^13^C-NMR of skimmianine, the characteristic three methoxy carbons were seen at δ 56.7, δ 58.8, and δ 61.5 ppm. The highest carbon chemical shift δ 164.2 ppm was observed for the methoxy group-attached carbon, which is present in the pyridine ring part of skimmianine. The other methoxy-attached carbons are seen at δ 152.0 and δ 157.0 ppm. The characteristic imine carbon C=N was observed at δ 142.1 ppm. All aromatic magnetically non-equivalent carbons are observed between δ 101.9 and δ 142.8 ppm (Figure S9, Table 4).

**Table 4:** NMR (^1^H-NMR and ^13^C-NMR) spectral data of skimmianine compound.

**Chemical shift (δ ppm)**	**Proton/carbon numbered**	**Splitting pattern**	**Nature of the proton/carbon**
^**1**^**H-NMR**			
4.03	3	Singlet	Methoxy
4.11	6	Singlet	Methoxy
4.41	7	Singlet	Methoxy
7.02	2	Doublet	C–H (Furan ring)
7.23	5	Doublet	C–H (Aromatic ring)
7.56	1	Doublet	C–H (Furan ring)
7.99	4	Doublet	C–H (Aromatic ring)
^**13**^**C-NMR**			
56.7	8	Singlet	Methoxy carbon
58.8	9	Singlet	Methoxy carbon
61.5	10	Singlet	Methoxy carbon
101.9	11	Singlet	Aromatic-fused ring carbon
104.4	2	Singlet	Furan-fused ring carbon
112.2	4	Singlet	Aromatic ring carbon
114.8	13	Singlet	Aromatic-fused ring carbon
117.9	5	Singlet	Aromatic ring carbon
141.4	14	Singlet	Aromatic-fused ring carbon
142.1	12	Singlet	Furan-fused ring carbon
142.8	1	Singlet	Furan ring carbon
152.0	6	Singlet	Aromatic ring carbon
157.0	7	Singlet	Aromatic ring carbon
164.2	3	Singlet	Aromatic ring carbon

### Antibacterial Activity of Isolated Compounds

Arborine and skimmianine compounds showed significant antibacterial activity against clinical isolated MDR *S. aureus* strains reported that 25 mm to 28mm of growth inhibition zones (Table 5). The results of the antibacterial activity of arborine and skimmianine clearly showed a dose-dependent effect (Table 6). The antibacterial activity results of arborine indicated that MDR *S. aureus* 101 and 410 strain was highly susceptible to the isolated compounds with the maximum inhibition of growth zone (28 mm) and lower MIC (0.2 μg/ml) and MBC (0.2 μg/ml) values. Similarly, skimmianine compound exhibit the highest antibacterial activity against the MDR *S. aureus* 315 strain (28 mm) with the lowest MIC (0.2 μg/ml) and MBC (0.2 μg/ml) values. The antibacterial activity was noticed in low concentration with stronger inhibitory potential. The results indicated that arborine and skimmianine exhibit a strong growth inhibition effect on all tested pathogens. The overall results indicated that the arborine and skimmianine possess better antibacterial activity than commercial antibiotics. Even a low MIC and MBC concentration of isolated compounds expressed a remarkable amount of bactericidal activity against the tested MDR *S. aureus* strains.

**Table 5:** Anti-bacterial activity of arborine and skimmianine compounds against MDR *S. aureus*.

**S. no**.	**MDR Strains**	**Arborine**	**Skimmianine**	**Amoxicillin**	**DMSO**
1	*S. aureus* 101	28 ± 0.58	27 ± 0.24	23 ± 0.58	00 ± 0.00
2	*S. aureus* 270	26 ± 0.00	25 ± 0.58	26 ± 0.58	00 ± 0.00
3	*S. aureus* 315	27 ± 0.58	28 ± 0.08	29 ± 0.58	00 ± 0.00
4	*S. aureus* 319	25 ± 0.15	26 ± 0.73	25 ± 0.58	00 ± 0.00
5	*S. aureus* 410	28 ± 0.39	25 ± 0.08	27 ± 0.58	00 ± 0.00

**Table 6:** MIC and MBC values of arborine and skimmianine compounds against MDR *S. aureus*.

**S. no**.	**MDR strains**	**MIC and MBC values (μg/ml)**					
		**Arborine**		**Skimmianine**		**Amoxicillin**	
		**MIC**	**MBC**	**MIC**	**MBC**	**MIC**	**MBC**
1	*S. aureus* 101	0.2	0.2	0.2	1	>2	>2
2	*S. aureus* 270	1	2	2	>2	2	>2
3	*S. aureus* 315	1	1	0.2	0.2	1	2
4	*S. aureus* 319	1	>2	1	1	>2	>2
5	*S. aureus* 410	0.2	0.2	2	>2	2	>2

### Bacterial Mechanism Study of Compounds

#### Time Kill Kinetic Assay

**Figure 2:**
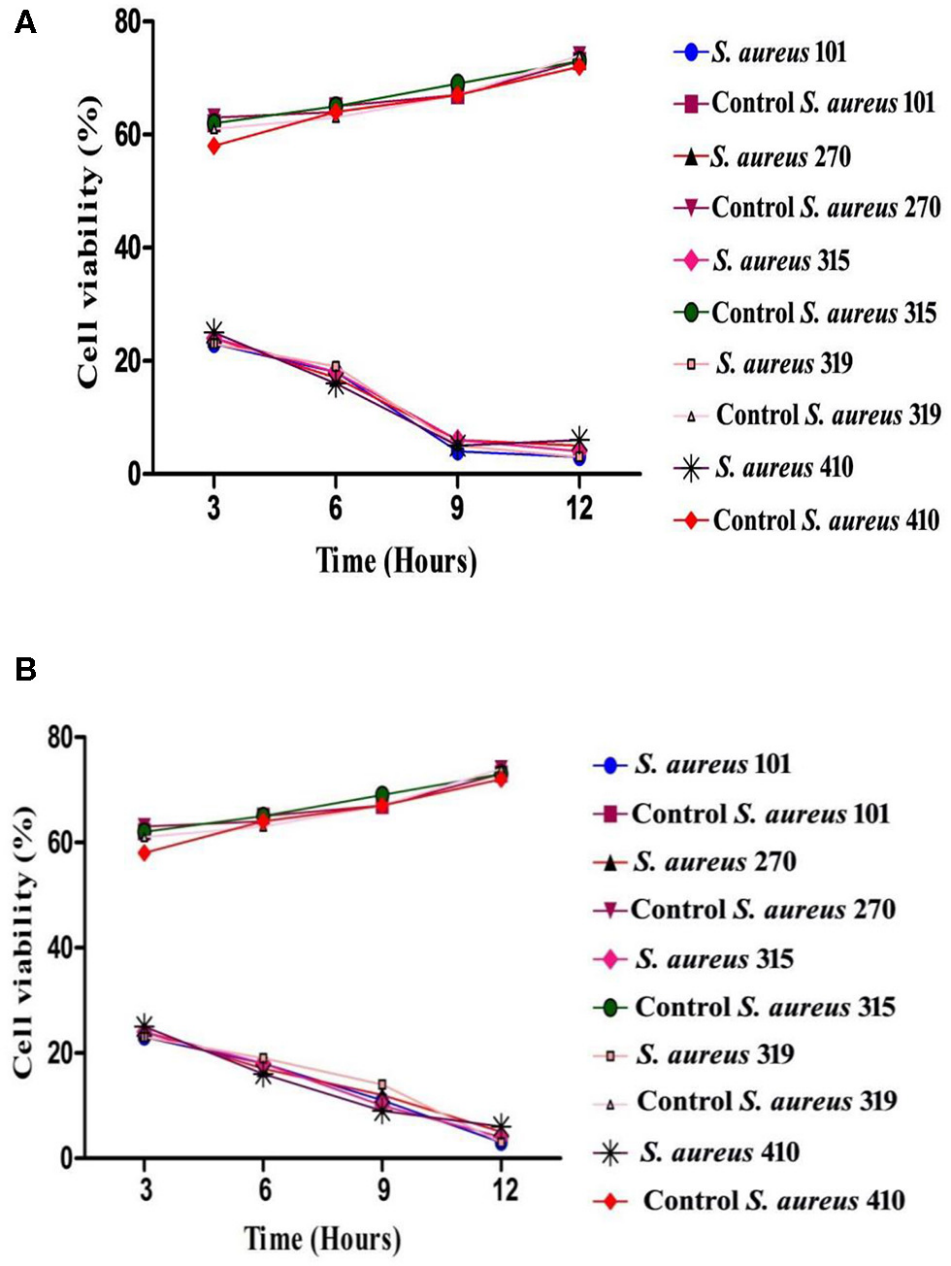
**(A)** Time-kill kinetic assay of arborine compound on different MDR *Staphylococcus aureus* strains. **(B)** Time-kill kinetic assay of skimmianine compound on different MDR *Staphylococcus aureus* strains.

The time-killing kinetic assay was used to analyze the post-treatment bacterial viability and to define the minimum time required to obtain the bactericidal effect. Both arborine and skimmianine compounds show similar time-killing kinetic patterns of bactericidal effect on the MDR *S. aureus* strains (Figures 2A,B). Bactericidal activity was gradually increased by the time up to 12 h exposure of the MDR *S. aureus* strains against arborine and skimmianine compounds at their respective MBC concentration for both the strains and the MDR *S. aureus* strains were killed within this period. The arborine and skimmianine compounds expressed a time-dependent and prompt bactericidal potential against the tested MDR *S. aureus* strains, which leads to bacterial death at the early stationary phase, as shown in time-kill curves (Figures 2A,B) compared with the control cells.

#### Protein Leakage Assay

**Figure 3:**
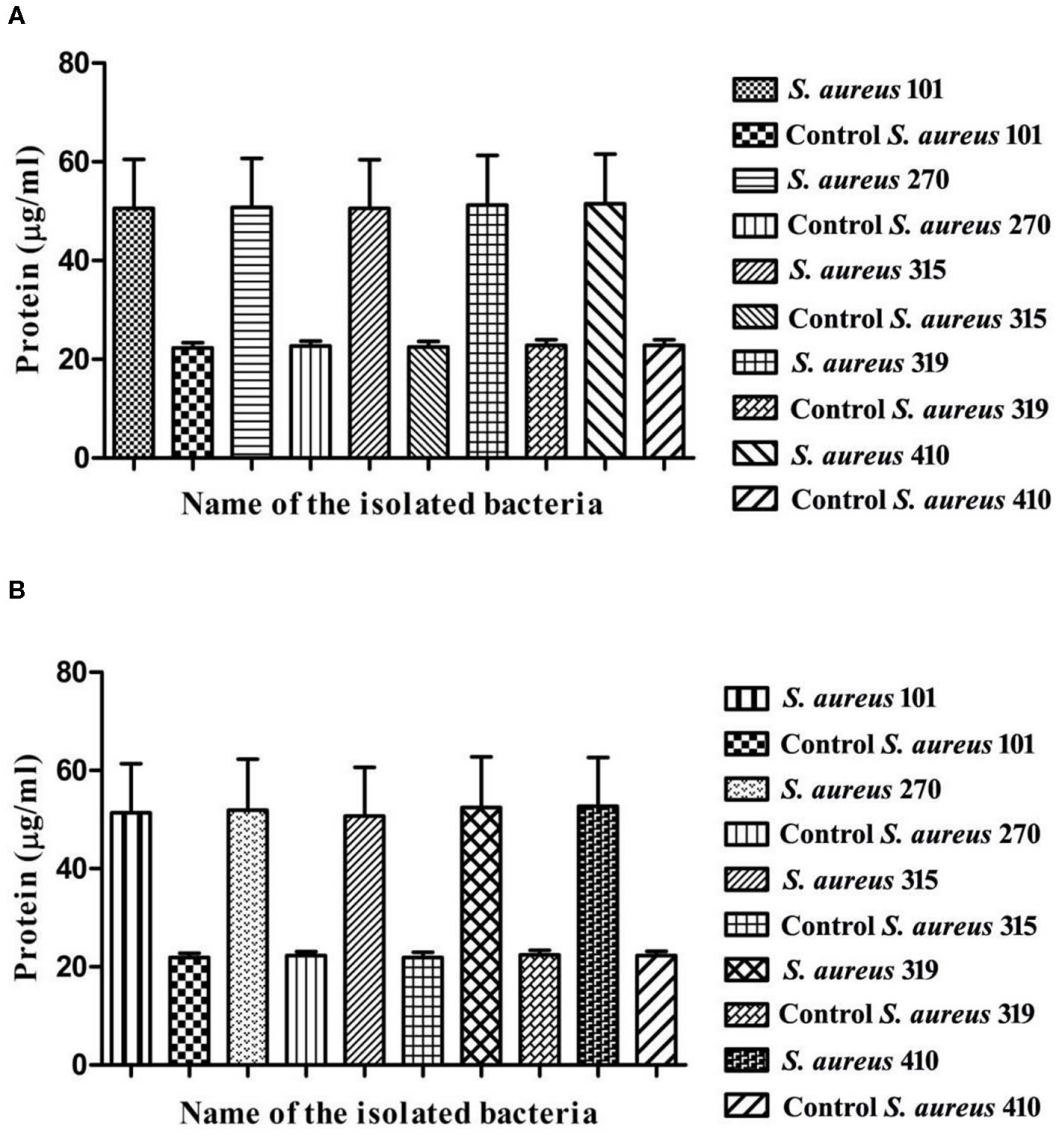
**(A)** Protein leakage assay of arborine compound on different MDR *Staphylococcus aureus* strains. **(B)** Protein leakage assay of skimmianine compound on different MDR *Staphylococcus aureus* strains.

Arborine and skimmianine compounds are known to enhance protein leakage by increasing the membrane permeability in MDR *S. aureus* strains. To determine the impact of arborine and skimmianine compounds alone on protein leakage, the cells were treated with arborine for 75 μg/ml, resulting in all strains exhibiting 54% protein leakage, and skimmianine compounds were treated for 100 μg/ml and the leakage of proteins was 55%. When the cells were treated with arborine and skimmianine compounds, the amount of protein released from the cells was increased compared to control (commercial antibiotics with recommended doses) (Figures 3A,B).

#### Morphological Changes of MDR S. aureus

**Figure 4:**
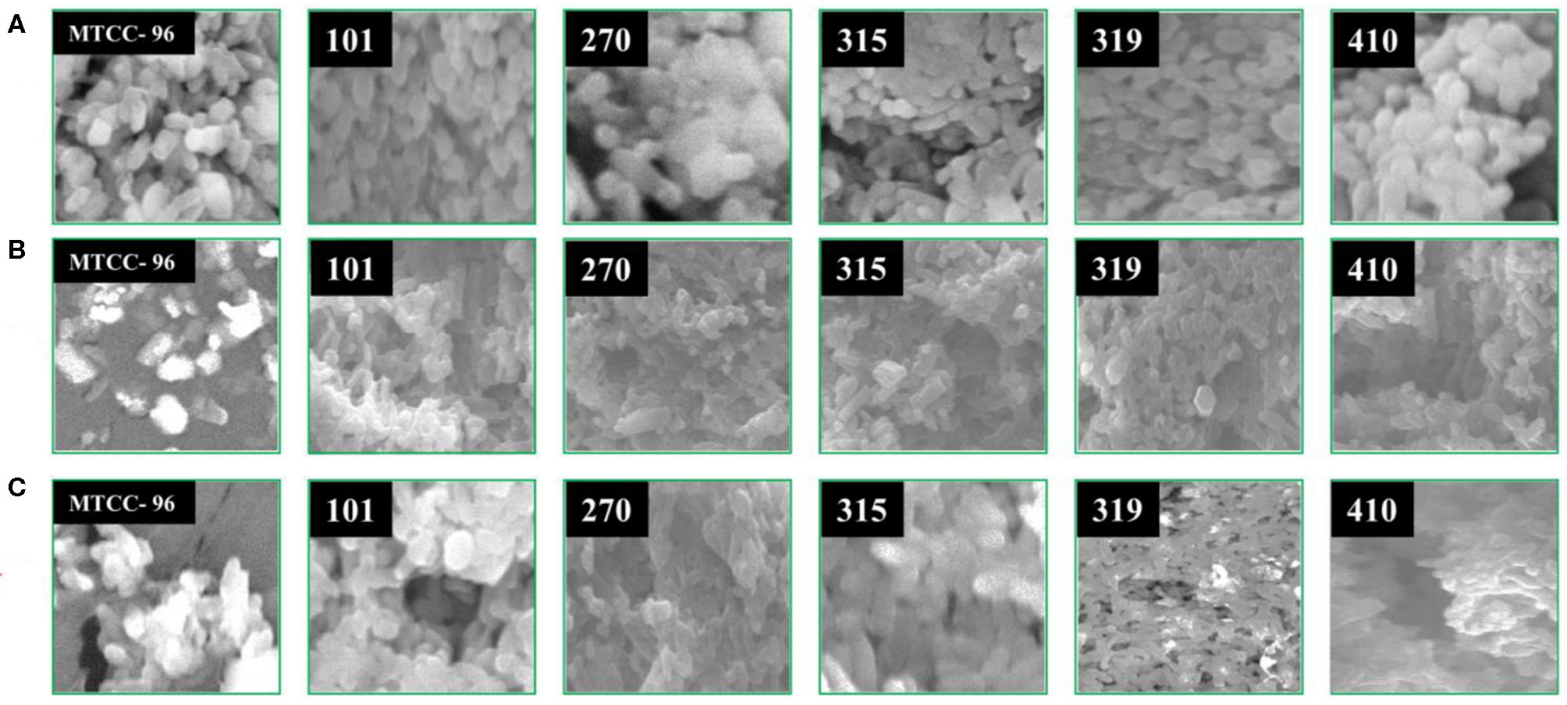
The effect of arborine and skimmianine on the morphology of different MDR *Staphylococcus aureus* strains observed under scanning electron microscopy. **(A)** Control (without any treatment), **(B)** arborine-treated, and **(C)** skimmianine*-*treated *S. aureus* cells (the numbers on the images represent the *S. aureus* strain numbers).

In order to have a better understanding of the antibacterial mechanism, arborine and skimmianine compounds were further studied for their effects on bacterial cell surfaces. Using SEM analysis, the present study observed the morphological changes in all MDR *S. aureus* strains and the reference standard *S. aureus* MTCC-96 strain, upon treatment with the antibacterial effect of arborine and skimmianine compounds (Figure 4). In MDR *S. aureus*, the untreated bacterial cells (control strains) appeared as grape-shaped (cocci), and the surfaces of the cells were intact with no damage observed. The *S. aureus* cells treated with arborine and skimmianine compounds, was not able to maintain the cocci (grape) shaped characteristics. Moreover, uneven fragments were observed, which indicated the damages induced on bacterial cell membranes. Moreover, the treated *S. aureus* cell surfaces were uneven, the size of cells were reduced, and the cells seemed to be damaged. Through the SEM analysis, the present study confirmed that the MDR *S. aureus* membrane surfaces were damaged, upon the treatment with arborine and skimmianine compounds isolated from *G. pentaphylla*.

## Discussion

Medicinal plants have been the major source for innumerable therapeutic agents, which are of great importance to the health of an individual as well as communities (30). *G. pentaphylla* ethyl acetate crude extract was separated into nine fractions by thin-layer chromatography. Only two fractions showed excellent antibacterial property. This fraction was composed of two compounds that were visualized in TLC under the UV chamber. This study clearly shows that the active fractions obtained from column chromatographic separation are found to be mixtures of compounds. The purity of the fractions was obtained by column chromatography. The column purified compounds were characterized by ^1^H NMR, C^13^ NMR, FT-IR, HPLC, and LC-MS. The compounds GP-1 and GP-2 exactly matched the structure of arborine and skimmianine compounds with the molecular formula C_16_H_14_N_2_O and C_14_H_13_NO_4_, respectively. The ^13^C NMR also supports the presence of the N-methyl and benzylic group. The characteristic functional groups found at 1602 cm^−1^ (C=O) and 1402 cm^−1^ (C–N) in the FT-IR spectra confirmed the arborine structure. Finally, the LC-MS also complies with the mass of arborine (MW 250), which is observed as an M+1 peak (251).

Similarly, Chakravarti et al. (31) have isolated the alkaloid arborine compound from the leaves of *Glycosmis arborea*, and its structure was further confirmed as 2-benzyl-1-methylquinazol-4-one based on UV, FT-IR, and ^I^HNMR analysis. The present findings correlated with the observations of the arborine compound isolated from the stem of *G. pentaphylla*, which was already reported by Chakravarti et al. (32), Govindachari et al. (33), and Muthukrishnana et al. (34). Ghani (35) reported that the *G. pentaphylla* leaves contain alkaloids arborine, arbornine, skimmianine, glycorine, glycophymine, glycophymoline, glycosmicine, and glycomide.

The structure of skimmianine was confirmed by ^1^H NMR, ^13^C NMR, FT-IR, and LC-MS. In ^13^C NMR, three methoxy carbons were observed at δ 56.7, 58.8, and 61.5 ppm. The FT-IR values of about 1,616 cm^−1^ (C=N) 1,266, 1,056 cm^−1^ (C–O, ether), and 1,056, 1,192 cm^−1^ (C-O, Furan) confirmed the structure of skimmianine. The LC-MS also confirmed the molecular weight of skimmianine, which was observed as M+1 peak (MW 260). The findings were in good agreement with earlier reports of Sreelekha (36) who isolated the skimmianine from *Zanthoxylum rhetsa*. Desai et al. (37) have isolated arborinine, cycleanine, isochondrodendrine, and skimmianine from the leaves of *G. pentaphylla*. Similarly, Sinhababu and Takur (38) have reported alkaloids arborine, arborinine, skimmianine, glycorine, glycosmicine, and an amide that was isolated from the flower heads of *G. pentaphylla*. On the other hand, Chakravarty et al. (39) isolated a skimmianine compound from *G. arborea*. Another study was done by Mester (40) and Greger et al. (41) isolated well-known alkaloids, arborine, and skimmianine compounds from *G. parviflora*.

The isolated arborine and skimmianine compounds show significant antibacterial activity against multidrug-resistant *S. aureus* isolates. Similar findings were reported by Bowen et al. (42), Chakravarti et al. (31), and Chakravarty et al. (39) who carried out the antibacterial activity of arborine and skimmianine compounds against both Gram-positive bacteria (*S. aureus* and MRSA) and Gram-negative bacteria (*E. coli* and *S. typhimurium*), which were inactive against *S. aureus*. Previously, Jeyachandran et al. (43) have reported that the plumbagin bioactive compound was isolated from the root extract of *Plumbago zeylanica*, exhibiting more toxic potential against *S. aureus*. The *in vitro* time-kill assay is one of the most commonly used experimental models to assess the antibacterial activity, efficiently characterizing the rate, extent, and timing of bacterial killing and regrowth (44). The time-kill test finds out the differences in the rate and extent of antibacterial activity over time, and it can also provide growth kinetics information (45). The overall result of this assay revealed a stronger bactericidal effect in arborine and skimmianine compounds against *S. aureus* than in a laboratory medium. The evaluation of protein leakage of the arborine and skimmianine showed a significant strong effect on the tested MDR *S. aureus* strains. A similar kind of protein leakage when treated with oil compounds from medicinal plants supports the findings of the present study (46).

The SEM result of the present study shows that bacterial cell membranes were significantly affected by the activity of arborine and skimmianine compounds. It is indicated that arborine and skimmianine bioactive compounds disturb the bacterial membrane and death to cells. Similar findings were reported by Campos et al. (47) who identified that the *S. aureus* and *E. coli* bacterial membranes and cytoplasm were highly affected due to the passive diffusion of plant metabolites into the cells that caused consequent cell disruption. These changes in bacteria cells may be due to the lysis of membrane and transformation caused by the damage on the permeability and integrity of membrane from arborine and skimmianine compounds. Consequently, the changes can lead to loss of inner cell materials (48, 49). The results of SEM were in good agreement with other findings of Paul et al. (50) and Sharma et al. (51) reported that other antimicrobials treated cells. Shen et al. (29) have reported that *S. aureus* damaged cell membrane affects the cell permeability, and outflow of cellular components that lead to cell death which supported the present study.

## Conclusion

The present study indicated that *G. pentaphylla* is a good source of arborine and skimmianine, which acts as potent antibacterial agents against MDR *S. aureus* clinical isolates. Besides, these compounds induced significant strong bactericidal effects on the MDR *S. aureus* such as intracellular molecular imbalance and cell membrane disturbances that caused cell death. Hence, this study recommends that the isolated compounds can be used as a template molecule for pharmaceutical drug design for the treatment of diseases caused by MDR *S. aureus*.

## Data Availability Statement

All datasets generated for this study are included in the article/Supplementary Material.

## Author Contributions

NM and RS equally contributed to the experiments and wrote the manuscript. AM, MK, and DN designed the research work and carry out the corrections in the article.

## Conflict of Interest

The authors declare that the research was conducted in the absence of any commercial or financial relationships that could be construed as a potential conflict of interest.
