# Supplementary material for: Glycosmis pentaphylla (Rutaceae): A Natural Candidate for the Isolation of Potential Bioactive Arborine and Skimmianine Compounds for Controlling Multidrug-Resistant Staphylococcus aureus
Source: Front Public Health. 2020 Jun 10;8:176. doi: 10.3389/fpubh.2020.00176 (PMC7297912; doi:10.3389/fpubh.2020.00176)
Supplement: Supplementary file 1 [file Data_Sheet_1.pdf]

SUPPLEMENTARY DATA

"*Glycosmis pentaphylla* (Rutaceae): A Natural candidate for the isolation of potential bioactive arborine and skimmianine compounds for controlling Multidrug Resistant *Staphylococcus aureus*"

Natarajan Murugan[1,†], Ramalingam Srinivasan[1,2,3,†], Athiappan Murugan[4], Myunghee Kim[2,¶], Devarajan Natarajan[1,*]

[1]Natural Drug Research Laboratory, Department of Biotechnology, School of Biosciences, Periyar University, Periyar Palkalai Nagar, Salem 636 011, Tamil Nadu, India

[2]Department of Food Science and Technology, Yeungnam University, Gyeongsan, Gyeongsangbuk-do 38541, Republic of Korea

[3]Department of Biotechnology, K. S. Rangasamy College of Arts and Science, K. S. R. Kalvi Nagar, Tiruchengode 637 215, Namakkal, Tamil Nadu, India

[4]Department of Microbiology, School of Biosciences, Periyar University, Periyar Palkalai Nagar, Salem 636 011, Tamil Nadu, India

[†]These authors contributed equally to this work.

Running Title: Arborine and Skimmianine for control of MDR *S. aureus.*

***Corresponding author:** Dr. Natarajan Devarajan, Department of Biotechnology, Periyar University, Periyar Palkalai Nagar, Salem 636 011, Tamil Nadu, India. Tel: +91-94-4385-7440; E-mail: natarajpu@gmail.com

¶Co-corresponding author: Dr. Myunghee Kim, Department of Food Science and Technology, College of Life and Applied Sciences, Yeungnam University, Gyeongsan-si, Gyeongsangbuk-do 38541, Republic of Korea. Tel.: +82-53-810-2958; Fax: +82 53-810-4662; E-mail: foodtech@ynu.ac.kr

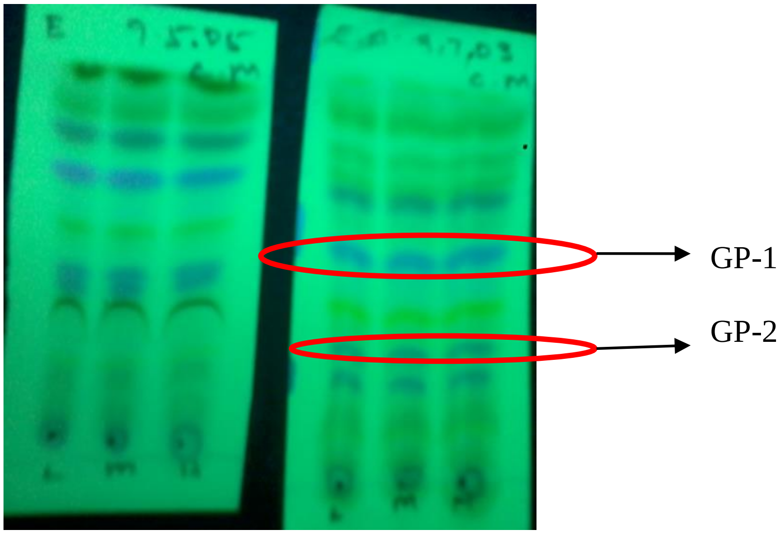

FIGURE S1. TLC chromatogram of ethyl acetate fraction of *G. pentaphylla*

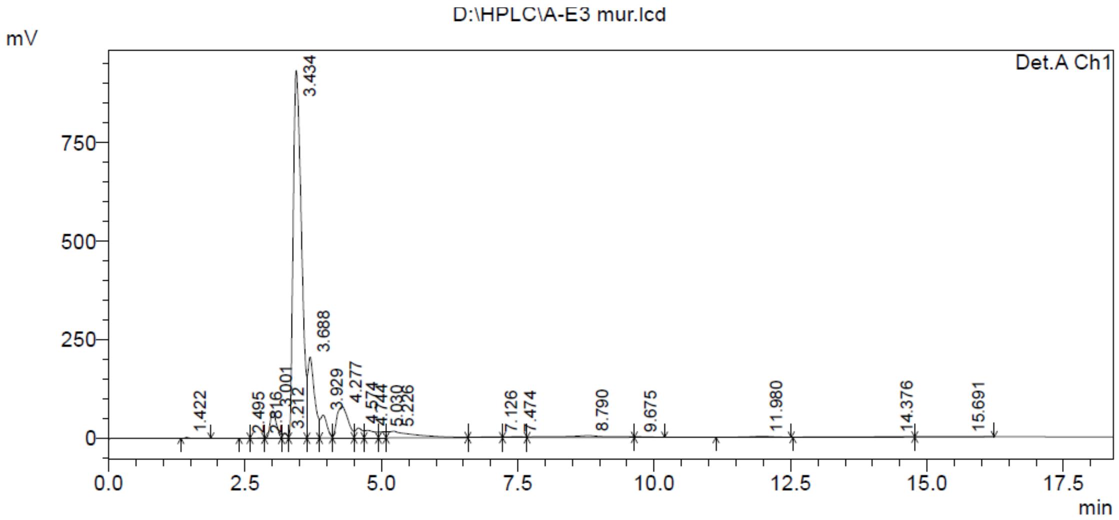

FIGURE S2. HPLC analysis of isolated compounds from ethyl acetate fraction of *G. pentaphylla*

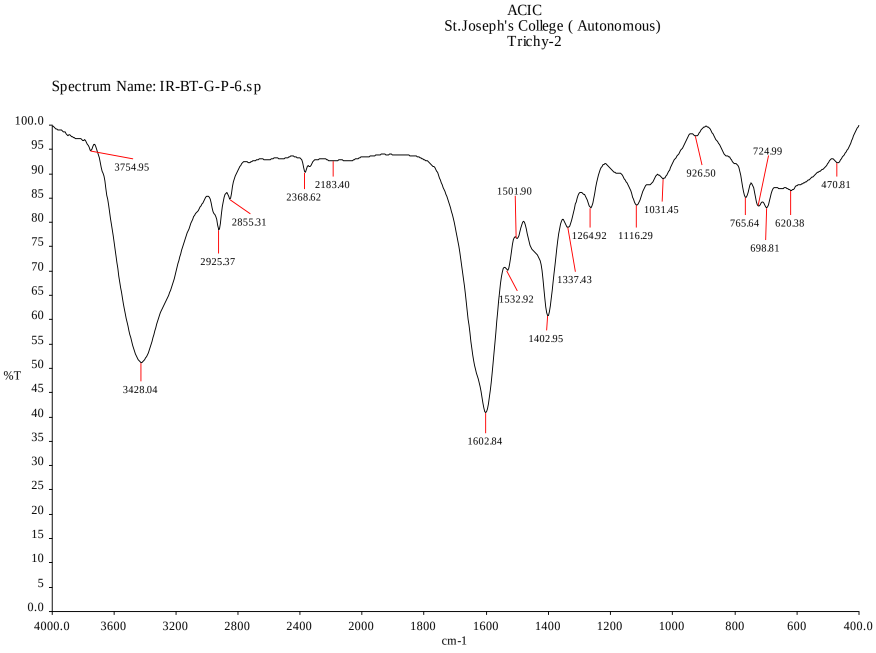

FIGURE S3. FT-IR spectrum of arborine

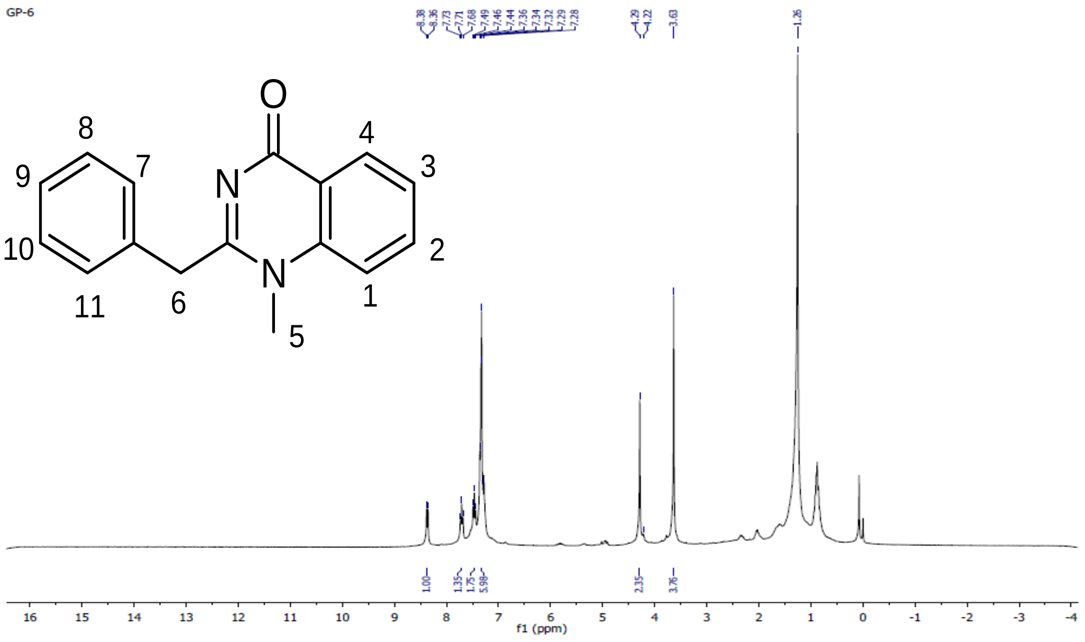

FIGURE S4. ^{1}H-NMR spectrum of arborine

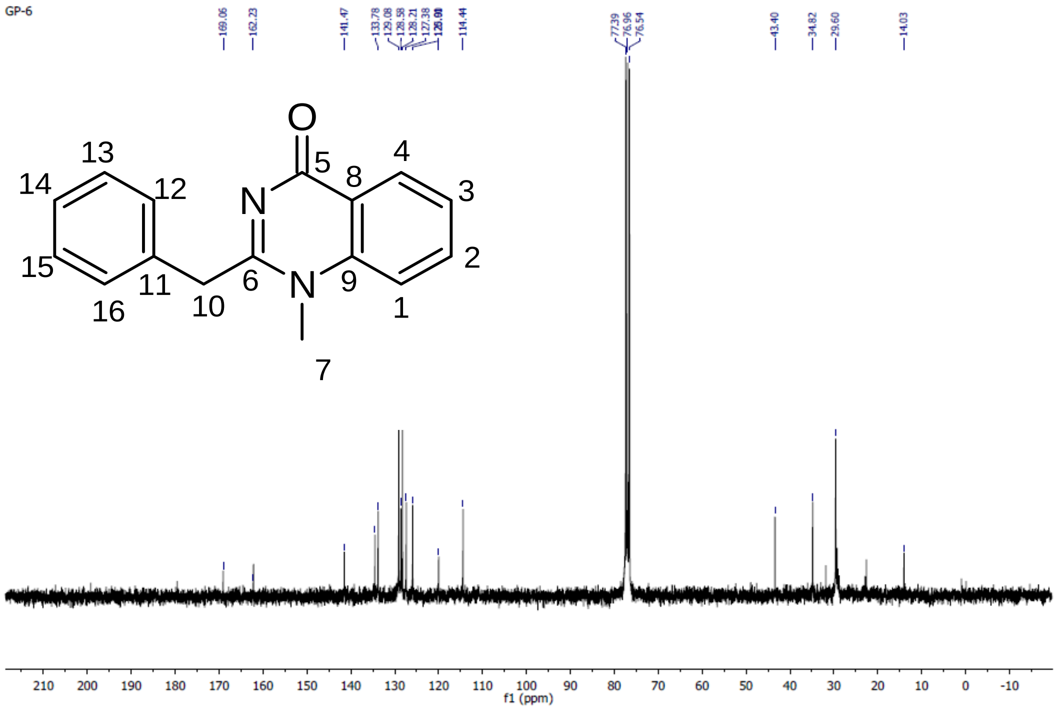

FIGURE S5. ^{13}C-NMR spectrum of arborine

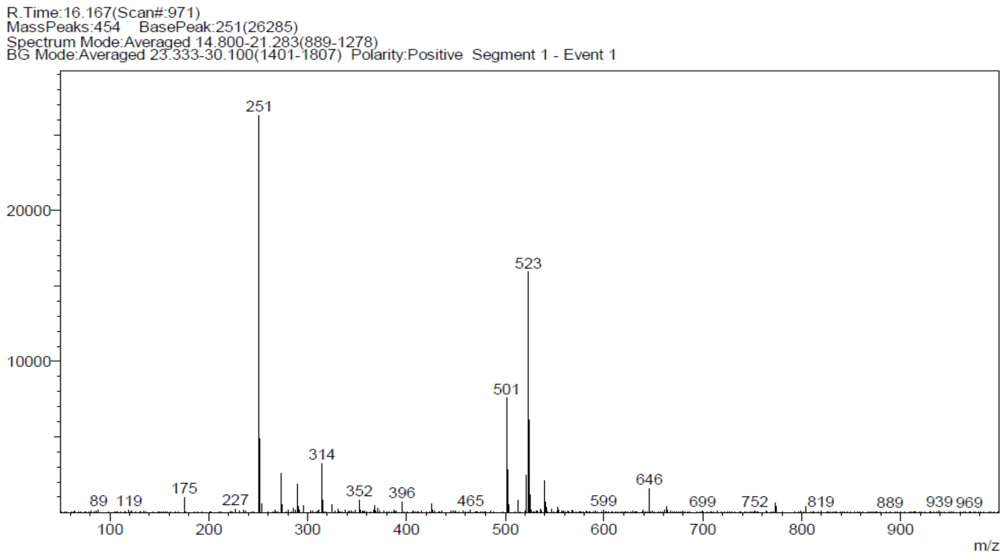

FIGURE S6. Mass spectrum of arborine

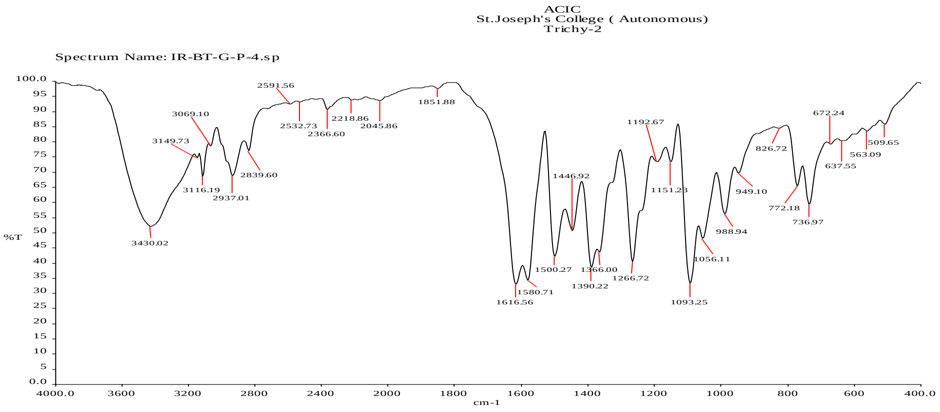

FIGURE S7. FT-IR spectrum of skimmianine

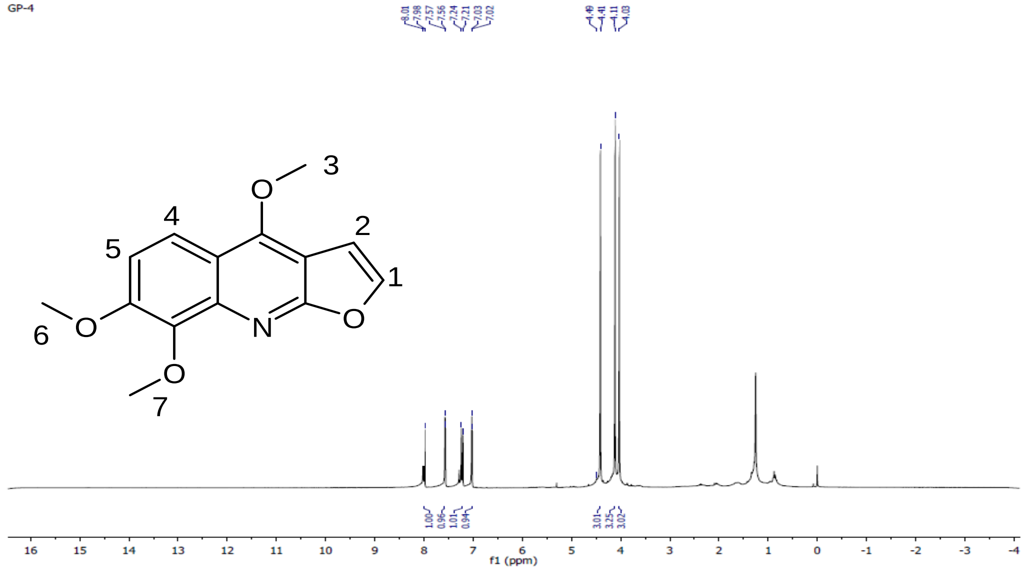

FIGURE S8. ^{1}H-NMR spectrum of skimmianine

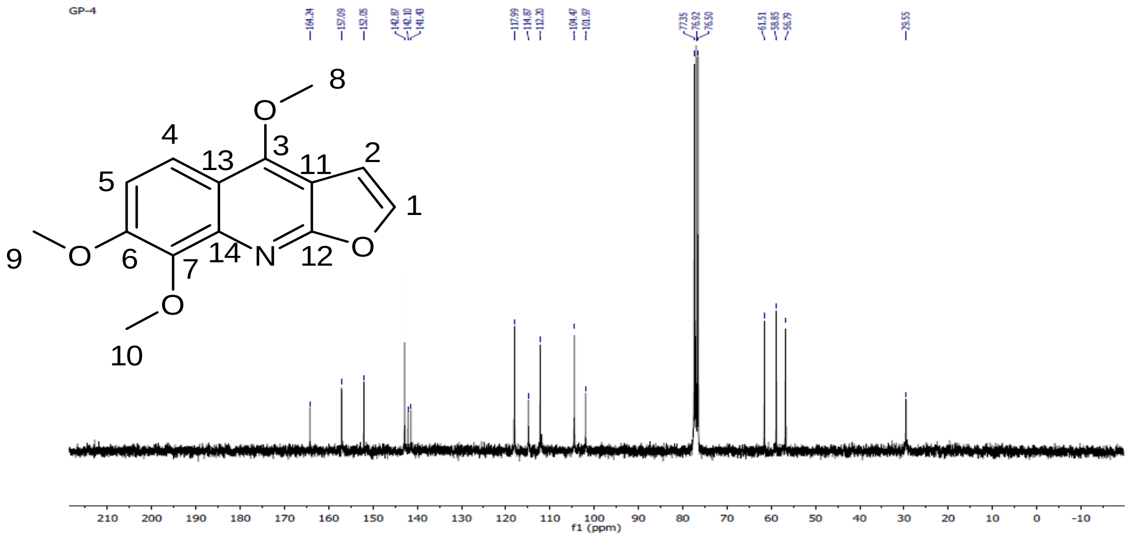

FIGURE S9. ^{13}**C-NMR spectrum of skimmianine**

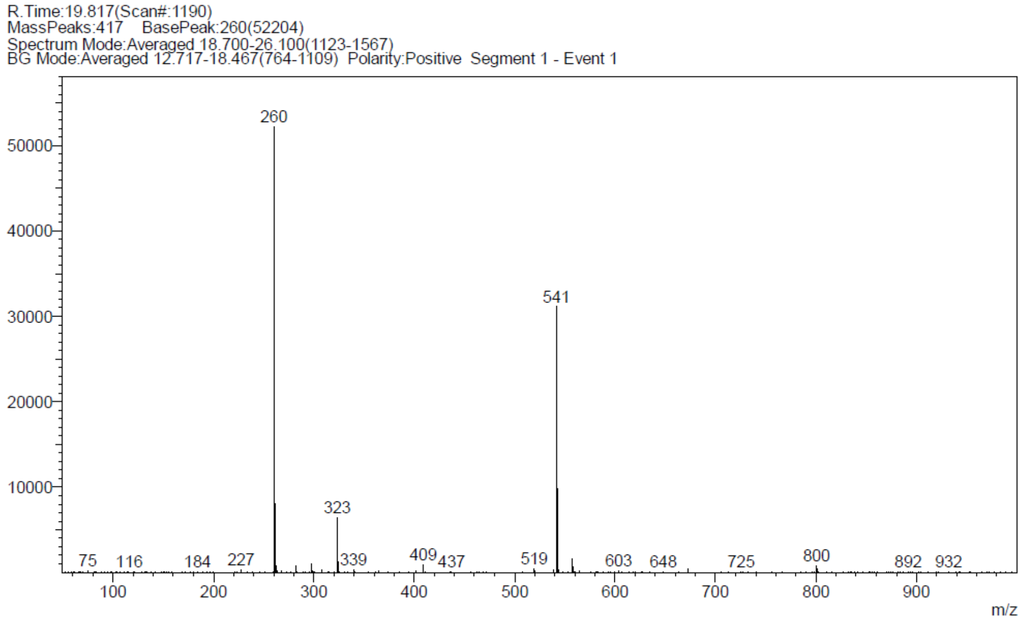

FIGURE S10. Mass spectrum of skimmianine
